# Co-opting ATP-generating glycolytic enzyme PGK1 phosphoglycerate kinase facilitates the assembly of viral replicase complexes

**DOI:** 10.1371/journal.ppat.1006689

**Published:** 2017-10-23

**Authors:** K. Reddisiva Prasanth, Chingkai Chuang, Peter D. Nagy

**Affiliations:** Department of Plant Pathology, University of Kentucky, Plant Science Building, Lexington, KY, United States of America; University of California, Davis Genome Center, UNITED STATES

## Abstract

The intricate interactions between viruses and hosts include exploitation of host cells for viral replication by using many cellular resources, metabolites and energy. Tomato bushy stunt virus (TBSV), similar to other (+)RNA viruses, induces major changes in infected cells that lead to the formation of large replication compartments consisting of aggregated peroxisomal and ER membranes. Yet, it is not known how TBSV obtains the energy to fuel these energy-consuming processes. In the current work, the authors discovered that TBSV co-opts the glycolytic ATP-generating Pgk1 phosphoglycerate kinase to facilitate the assembly of new viral replicase complexes. The recruitment of Pgk1 into the viral replication compartment is through direct interaction with the viral replication proteins. Altogether, we provide evidence that the ATP generated locally within the replication compartment by the co-opted Pgk1 is used to fuel the ATP-requirement of the co-opted heat shock protein 70 (Hsp70) chaperone, which is essential for the assembly of new viral replicase complexes and the activation of functional viral RNA-dependent RNA polymerase. The advantage of direct recruitment of Pgk1 into the virus replication compartment could be that the virus replicase assembly does not need to intensively compete with cellular processes for access to ATP. In addition, local production of ATP within the replication compartment could greatly facilitate the efficiency of Hsp70-driven replicase assembly by providing high ATP concentration within the replication compartment.

## Author summary

Positive-strand (+)RNA viruses build replication organelles with the help of many co-opted cellular factors and by usurping cellular metabolites and energy, which frequently lead to disease states in plants, animals and humans. The authors discovered that tomato bushy stunt virus (TBSV) co-opts the glycolytic ATP-generating Pgk1 phosphoglycerate kinase to facilitate the intracellular assembly of new viral replicase complexes. The ATP generated by co-opted Pgk1 within the replication compartment is used to fuel the ATP requirement of co-opted cellular heat shock protein 70 chaperone, which is essential for the assembly of new TBSV replicase complexes. Direct recruitment of Pgk1 by TBSV might provide easy access to ATP. Metabolic reprogramming of the glycolytic pathway might be a widespread phenomenon during various viral infections.

## Introduction

Positive-strand (+)RNA viruses build robust viral replication machineries, called replication organelles, with the help of many co-opted cellular factors. In addition, viruses also obtain metabolites and energy from the infected cells. Overall, (+)RNA viruses induce major metabolic and structural changes in the infected cells, which frequently lead to disease states [1,2,3,4,5].

One of the best characterized (+)RNA virus is TBSV, which induces large replication compartments consisting of aggregated peroxisomal and ER membranes. The replication compartments contain numerous vesicle-like structures in the limiting membrane of peroxisomes, which harbor the viral replicase [6,7,8]. TBSV also manipulates the cytoskeleton and endosomal trafficking [9,10]. TBSV co-opts numerous host proteins to support various viral functions, including the heat shock protein 70 (Hsp70), the endosomal sorting complex required for transport (ESCRT) machinery, translation factors and DEAD-box RNA helicases [11,12,13,14,15,16,17]. TBSV also induces enrichment of sterols at the viral replication sites via co-opting oxysterol-binding proteins and membrane contact sites and retargets phosphatidylethanolamine to the replication sites to build suitable membranous subcellular environment for replication [8,9,18]. TBSV-induced major changes in the cell require energy, but how TBSV can obtain the energy to fuel these energy-demanding processes is not known.

Glycolysis is an essential and highly conserved energy-producing pathway in the cytosol. Glucose is converted into pyruvate by a ten-enzyme catalyzed reaction that produces ATP and NADPH. The two ATP-generating enzymes in the glycolytic pathway are Pgk1 phosphoglycerate kinase and Cdc19 pyruvate kinase (PKM2/PKLR in humans). Pgk1 produces ATP from ADP and DPG using substrate-level phosphorylation in the cytosol [19]. Pgk1 is highly conserved and present in every organism. In humans, Pgk1 is also important for tumor growth and DNA replication/repair and mutations in Pgk1 are associated with myopathy, mental disorders and hemolytic anemia [20,21,22]. Pgk1 is also important for the activation of nucleotide-based anti-HIV drugs [23].

Previous genome-wide screens in yeast model host have identified several components of the glycolytic pathway that affected TBSV replication [24,25,26]. Proteomic screens also identified Pgk1 phosphoglycerate kinase, which was pulled-down with the tombusviral p92 RdRp and interacted with p33 replication protein [27,28]. Therefore, we decided to characterize the putative role of Pgk1 in tombusvirus replication in this work. We discovered that TBSV co-opts the glycolytic Pgk1 through direct interaction with the viral replication proteins. Altogether, we provide evidence that the ATP generated locally in the viral replication compartment by the co-opted Pgk1 is used to fuel the ATP requirement of the co-opted cellular Hsp70 molecular chaperones. The functions of co-opted Hsp70 are to facilitate the assembly of new viral replicase complexes and the activation of the viral RNA-dependent RNA polymerase. The local production of ATP by Pgk1 within the replication compartment could greatly facilitate the efficiency of Hsp70-driven replicase assembly by providing high ATP concentration within the replication compartment.

## Results

### Recruitment of the cytosolic Pgk1 phosphoglycerate kinase into the tombusvirus replication compartment in yeast and plant cells

**Fig 1:**
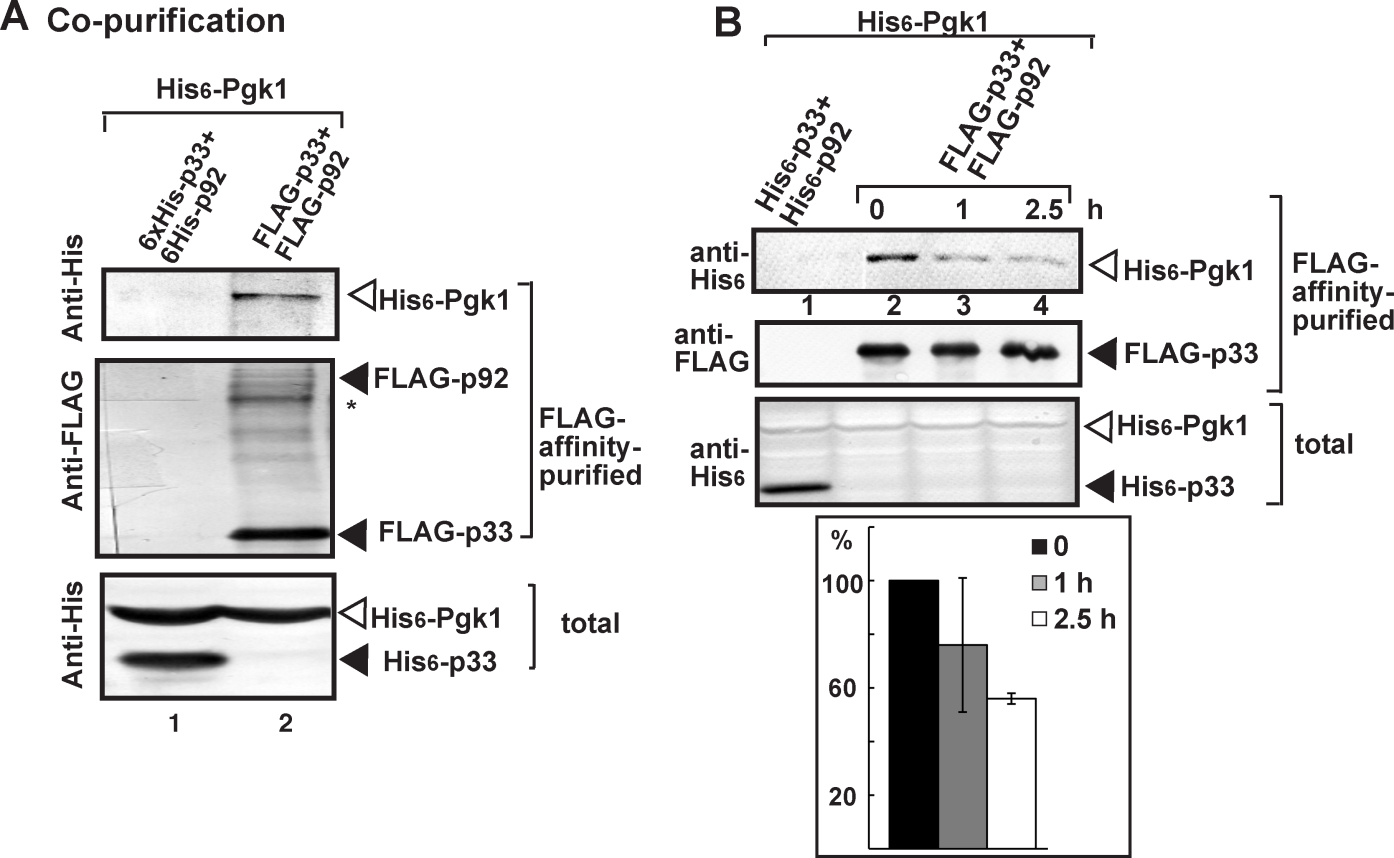
Interaction between p33 and p92^pol^ replication proteins and yeast cytosolic Pgk1. (A) Co-purification of Pgk1 with the viral replicase. Top panel: Western blot analysis of co-purified His_6_-tagged Pgk1 with FLAG-affinity purified FLAG-p33 and FLAG-p92^pol^ from membrane fraction of yeast. Pgk1 was detected with anti-His antibody. The negative control was His_6_-tagged p33 and His_6_-p92 purified from yeast extracts using a FLAG-affinity column. Middle panel: Western blot of purified FLAG-p33 and FLAG-p92^pol^ detected with anti-FLAG antibody. Asterisk marks the SDS-resistant p33 homodimer. Bottom panel: Western blot of His_6_-tagged Pgk1 and His_6_-p33 (lane 1) proteins in the total yeast extracts using anti-His antibody. (B) Testing the presence of Pgk1 in the membrane-bound viral replicase after blocking cellular translation by cycloheximide. Top panel: Western blot analysis shows the co-purified His_6_-tagged Pgk1 with the viral replicase isolated from membrane fraction at the shown time points. See further details in panel A. Middle panel: Western blot analysis of the purified FLAG-p33 with anti-FLAG antibody. Bottom panel: Western blot analysis of His_6_-Pgk1 in the total yeast lysates with anti-His antibody. Each experiment was repeated three times.

Among the surprises from high-throughput screens with TBSV and yeast model host were the identification of the glycolytic Pgk1 as a putative co-opted host factor in TBSV replication [24,25,26,29]. To demonstrate interaction between Pgk1 and the viral replication proteins, first we performed co-purification experiments from yeast replicating TBSV repRNA. The isolated membrane fraction was solubilized with detergent and the FLAG-tagged p33 and FLAG-p92^pol^ replication proteins were affinity-purified, followed by Western-blot analysis. We found that the His_6_-tagged Pgk1 expressed from a plasmid was co-purified with the tombusvirus replicase (Fig 1A, lane 2). In contrast, Pgk1 was not co-purified with Flag-GFP control from yeast (S1 Fig). To examine if Pgk1 is a permanent component of the tombusvirus replicase, we halted the formation of new tombusvirus replicase complexes by shutting down p33 and p92^pol^ expression and stopping ribosomal translation by adding cycloheximide [7]. FLAG-affinity-purification of the tombusvirus replicase from the membrane fraction of yeast at various time-points showed the rapid release of Pgk1 from the replicase (Fig 1B, lanes 3–4 versus 2). Thus, Pgk1 seems to be a temporarily co-opted factor in the tombusvirus replicase complex.

**Fig 2:**
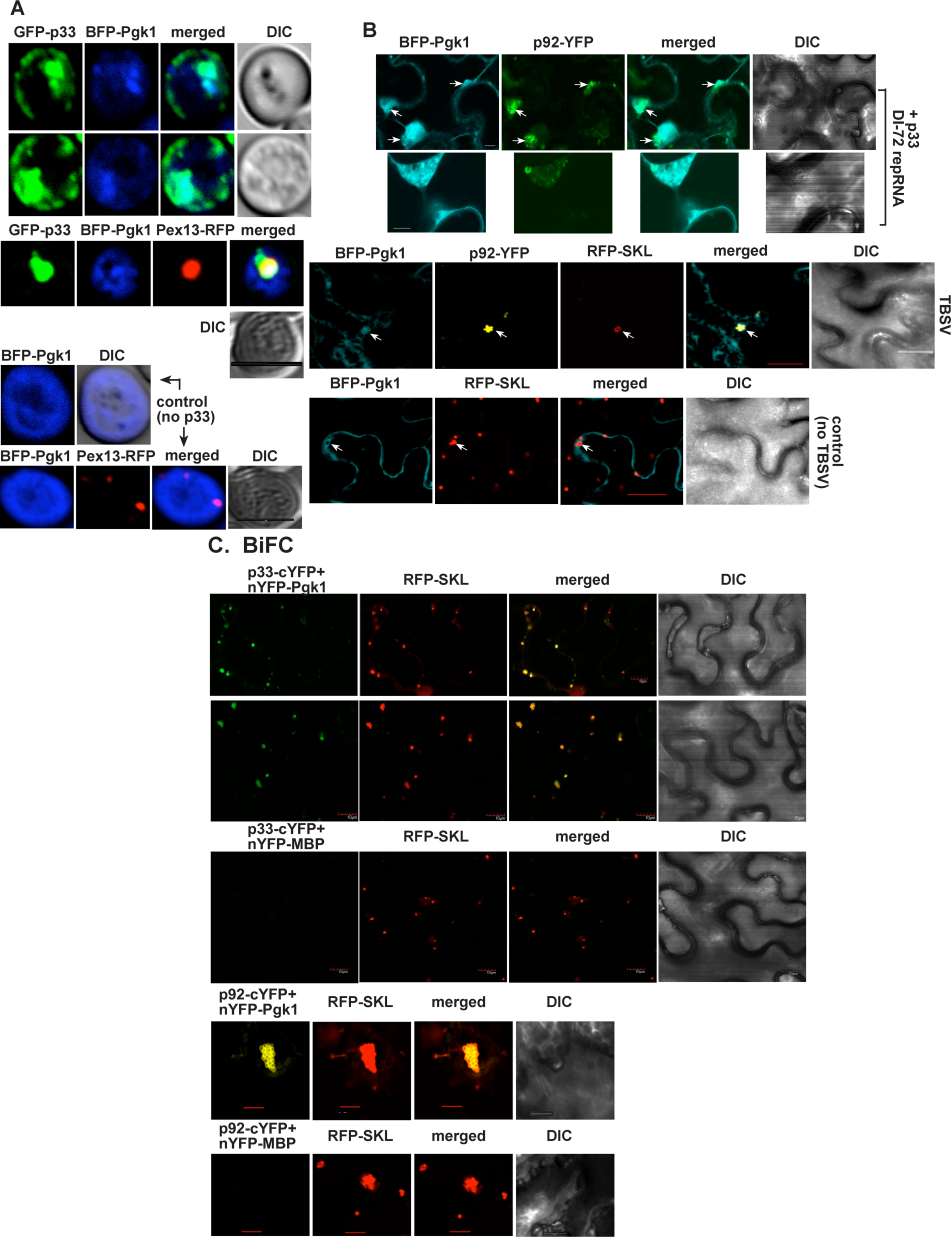
Recruitment of Pgk1 into the viral replication compartment in yeast and plant cells. (A) Confocal laser microscopy images show the partial co-localization of TBSV GFP-tagged p33 with the BFP-tagged Pgk1 protein in wt yeast cells. DIC (differential interference contrast) images are shown on the right. Bottom images show the expected cytosolic distribution of BFP-Pgk1 in the absence of viral components in a wt yeast cell. Pex13-RFP is expressed as a peroxisomal marker in experiments presented as the third and fifth panels. Scale bars represent 5 μm. (B) Confocal laser microscopy shows partial co-localization of TBSV YFP-tagged p92^pol^ replication protein with the BFP-NbPgk1 protein in *N*. *benthamiana* cells. Expression of the above proteins together with the untagged p33 and the DI-72 RNA from the 35S promoter was achieved after co-agroinfiltration of *N*. *benthamiana* leaves. The large structures represent the viral replication compartments. Bottom image: in the control experiment, the BFP-NbPgk1 protein shows the expected cytosolic distribution in the absence of viral components in *N*. *benthamiana* cells. Scale bars represent 5 μm (second panel), 10 μm (top panel), and 20 μm (bottom two panels), respectively. (C) Top two panels: *In planta* interaction between of TBSV p33-cYFP replication protein and the nYFP-NbPgk1 protein. Expression of the above proteins from the 35S promoter was done after co-agroinfiltration into *N*. *benthamiana* leaves. Note that p33-cYFP and the nYFP-NbPgk1 proteins were detected by BiFC. The interaction between p33 replication protein and NbPgk1 occurs in the replication compartment decorated by RFP-SKL (peroxisomal luminar marker). Third panel: control BiFC experiments included nYFP-MBP protein in combination with p33-cYFP (bottom panel). Fourth panel: *In planta* interaction between TBSV p92-cYFP replication protein and the nYFP-NbPgk1 protein. Fifth panel: control BiFC experiments included nYFP-MBP protein in combination with p92-cYFP. Scale bars represent 10 μm (top three panels), and 20 μm (bottom two panels), respectively.

To obtain additional evidence of subversion of Pgk1 into the tombusvirus replication compartment, we performed confocal laser microscopy experiments in yeast expressing GFP-p33 and BFP-Pgk1. Interestingly, we observed the robust recruitment of the cytosolic Pgk1 into the tombusvirus replication compartment, which was marked by Pex13-RFP peroxisomal marker protein (Fig 2A). Similar experiments with ectopically expressed BFP-NbPgk1 and p92-YFP showed the enrichment of Pgk1 in the tombusvirus replication compartment in *Nicotiana benthamiana* cells replicating the TBSV repRNA (Fig 2B). In addition, bimolecular fluorescence complementation (BiFC) assay further confirmed the recruitment of the glycolytic NbPgk1 via interaction with p33 into the peroxisomal TBSV replication compartment (Fig 2C). Similarly, we observed that the interaction between TBSV p92^pol^ replication protein and the glycolytic NbPgk1 takes place in the large replication compartment, which was marked by RFP-SKL peroxisomal luminal marker protein (Fig 2C, lower panels). Therefore, we conclude that TBSV recruits the cytosolic Pgk1 through direct interaction with the viral replication proteins into the viral replication compartment in both yeast and plant cells.

### Pgk1 acts as a pro-viral host factor in yeast

**Fig 3:**
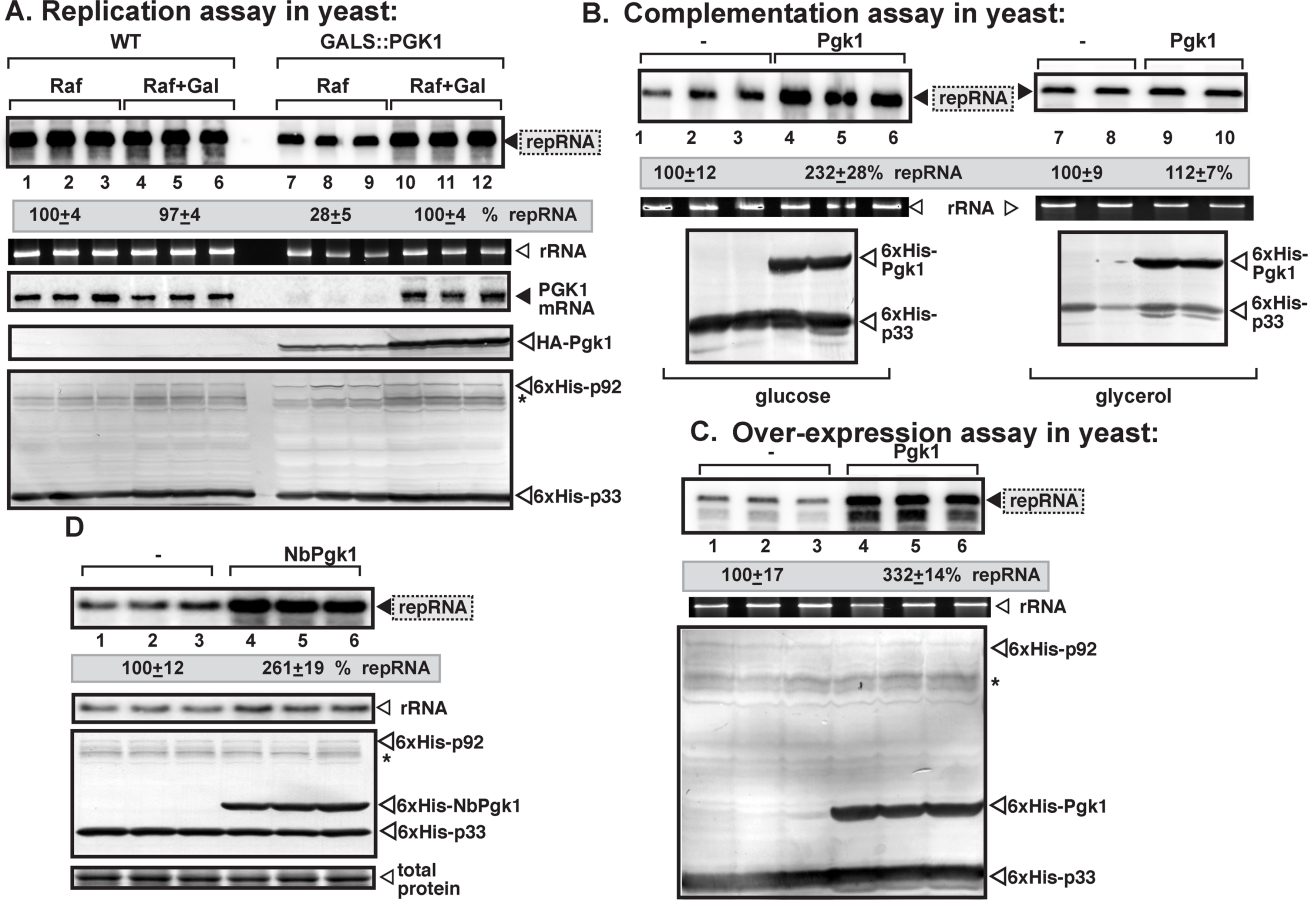
Reduced TBSV repRNA accumulation in yeast with depleted Pgk1 level. (A) Northern blot analysis shows decreased TBSV repRNA accumulation in a yeast strain (GALS::PGK1) when Pgk1 was depleted. To launch TBSV repRNA replication, we expressed His_6_-p33 and His_6_-p92^pol^ from the copper-inducible *CUP1* promoter, and DI-72(+) repRNA from the *ADH1* promoter in the parental (BY4741) and in GALS::PGK1 yeast strains. Note that GALS::PGK1 yeast strain expresses HA-tagged Pgk1 from the galactose-inducible *GALS* promoter from chromosomal location (i.e., HA-Pgk1 replaces the wt Pgk1 in this haploid yeast strain). The yeast cells were cultured for 16 hours at 29°C in either 2% galactose +2% raffinose [(Raf+Gal), inducing condition for HA-Pgk1] or 2% raffinose (lack of induction of HA-Pgk1 expression) SC minimal media supplemented with 50 μM CuSO_4_. The accumulation level of DI-72(+) repRNA was normalized based on 18S rRNA levels (second panel from top). Middle panel: Northern blot analysis of *PGK1* mRNA levels in total RNA samples. Bottom two panels: Western blot analysis of the accumulation level of HA-tagged Pgk1, His_6_-tagged p33, His_6_-p92^pol^ proteins using anti-HA and anti-His antibodies, respectively. Each experiment was performed three times. (B) Complementation assay with plasmid-borne expression of His_6_-Pgk1 in GALS::PGK1 yeast strain. His_6_-Pgk1 was expressed from the *TEF1* constitutive promoter, whereas the expression of the chromosomal HA-tagged Pgk1 was suppressed via 2% glucose in the growth media. Top panels: Northern blot analysis of repRNA level, middle panel: ethidium-bromide stained gel with ribosomal RNA, as a loading control, whereas bottom panels show Western blot analysis using anti-His antibody. Panels on the right represent samples obtained from yeast grown on the nonfermentable glycerol media. (C) The effect of over-expression of yeast Pgk1 on TBSV repRNA accumulation. The plasmid-borne His_6_-Pgk1 was expressed from *TEF1* promoter in BY4741 (wt) yeast. See further details in panel B. (D) The effect of heterologous expression of NbPgk1 on TBSV repRNA accumulation in yeast. The plasmid-borne His_6_-NbPgk1 was expressed from *TEF1* promoter in BY4741 (wt) yeast. See further details in panel B.

To test if the recruitment of Pgk1 into the viral replication compartment plays a role in TBSV replication, we have made a haploid yeast strain, in which the wt *PGK1* gene was replaced with the HA-tagged *PGK1*. In addition, expression of HA-*PGK1* was placed under the regulation of *GALS* promoter, thus allowing induction by the addition of galactose and repression by addition of glucose to the culture media [30]. Induction of TBSV repRNA replication in GALS::PGK1 yeast with suppressed *PGK1* expression showed ~4-fold reduction in TBSV repRNA replication (Fig 3A, lanes 7–9 versus 10–12, and S2 Fig). Expression of Pgk1 from a plasmid in GALS::PGK1 yeast with suppressed Pgk1 expression from chromosome increased TBSV repRNA replication by ~2-fold in glucose-containing media, while did not increase it on the non-fermentable glycerol-media (Fig 3B, lanes 7–10). These data indicate that Pgk1 plays a pro-viral function during TBSV replication in yeast.

Over-expression of either yeast Pgk1 (Fig 3C) or the *N*. *benthamiana* cytosolic NbPgk1 (Fig 3D) led to ~3-fold and a ~2.5-fold increase, respectively, in TBSV repRNA accumulation in wt yeast, confirming a pro-viral function for Pgk1 in TBSV replication.

### Pgk1 also acts as a pro-viral host factor in plant cells

**Fig 4:**
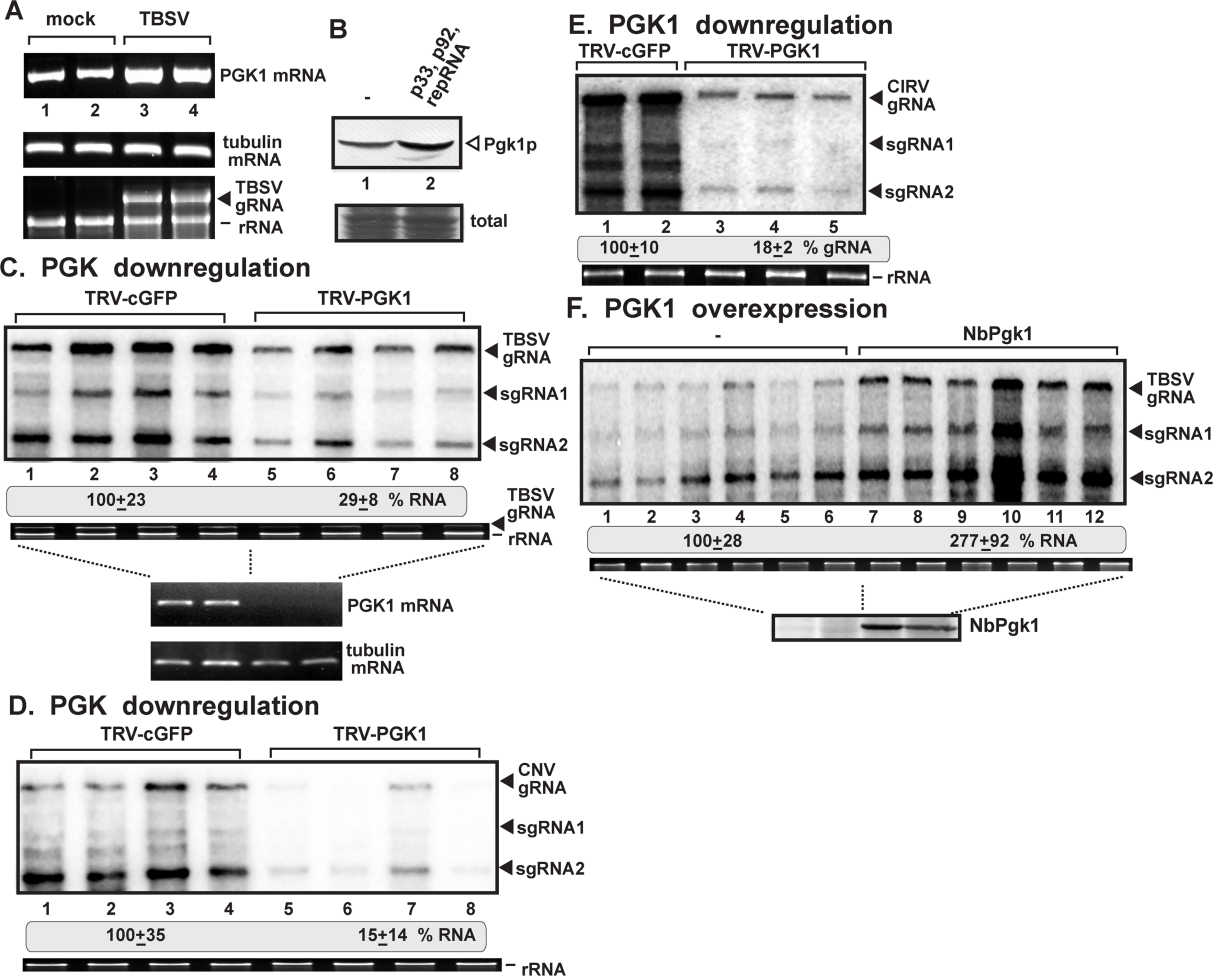
The pro-viral role of cytosolic Pgk1 in tombusvirus replication in *N*. *benthamiana*. (A) Up-regulation of Pgk1 expression in TBSV-infected *N*. *benthamiana* leaves. The mRNA levels for the cytosolic Pgk1 were estimated by semi-quantitative RT-PCR (25 and 28 cycles, the latter is shown) in total RNA samples obtained from either TBSV or mock-infected *N*. *benthamiana* leaves. Tubulin mRNA was used as a control (second panel). The bottom panel shows an ethidium-bromide stained agarose gel of total RNA samples with ribosomal RNA and TBSV genomic (g)RNA. (B) Western blot analysis of cytosolic Pgk1 level in total protein samples obtained from yeast either supporting TBSV repRNA accumulation or TBSV-free using anti-Pgk1 antibody. (C-E) Knock-down of *PGK1* mRNA level by VIGS inhibits the accumulation of tombusvirus RNA in *N*. *benthamiana*. Top panels: Total RNA samples obtained from *N*. *benthamiana* leaves silenced as shown were analyzed by Northern blotting, which shows the accumulation of TBSV gRNA and sgRNAs in panel C, the closely-related cucumber necrosis virus (CNV) RNAs in panel D and the mitochondria-replicating CIRV RNAs in panel E. Bottom images: ethidium-bromide stained gels show ribosomal RNA level. We chose the 12th day after VIGS to inoculate the upper, systemically-silenced leaves with TBSV virions, or agroinfiltrate with pGD-CNV or pGD-CIRV. Samples for RNA extractions were taken 1 day (TBSV) and 2.5 days (CNV or CIRV) post inoculation from the inoculated leaves. The control experiments included the TRV2-cGFP vector. Each experiment was performed three times. (F) Over-expression of the cytosolic NbPgk1 was done in *N*. *benthamiana* leaves by agroinfiltration. The same leaves, which were first agroinfiltrated with pGD-NbPgk1 (expressing NbPgk1 from the 35S promoter), were also inoculated with TBSV virions 2.5 days later. Then, total RNA samples were obtained the subsequent day (1 dpi). The control samples were obtained from leaves agroinfiltrated with pGD empty vector (not expressing proteins) (lanes 1–6). Northern blotting was used to detect the accumulation of TBSV RNAs in total RNA samples obtained from *N*. *benthamiana* leaves. The ribosomal RNA (rRNA) was used as a loading control and shown in agarose gel stained with ethidium-bromide (bottom panel). The bottom image shows a representative Western blot-based detection of His_6_-tagged NbPgk1 using anti-His antibody. Each experiment was performed three times.

Estimation of *PGK1* mRNA levels in TBSV-infected *N*. *benthamiana* leaves indicated ~2-fold increased level of expression (Fig 4A). Similarly, Western blotting demonstrated a ~50% increase in accumulation of Pgk1 protein in yeast cells replicating TBSV repRNA (Fig 4B), suggesting that a tombusvirus can mildly induce the accumulation of a glycolytic enzyme in cells.

To confirm the importance of Pgk1 in TBSV replication in a plant host, we knocked-down via virus-induced gene silencing (VIGS) the expression of the cytosolic Pgk1 in *N*. *benthamiana* and the Pgk1-silenced leaves were inoculated with the full-length infectious TBSV. TBSV genomic RNA accumulation was inhibited by ~4-fold in these Pgk1 knock-down plants (Fig 4C). Inoculation of Pgk1-silenced *N*. *benthamiana* with the similar peroxisome-replicating cucumber necrosis virus (CNV) (S3 Fig) and the mitochondrial-replicating carnation Italian ringspot virus (CIRV) showed a ~6-fold decrease in the accumulation of genomic RNAs (Fig 4D and 4E). Thus, the cytosolic Pgk1 is a critical pro-viral host factor for various tombusviruses in a plant host.

Over-expression of NbPgk1 in *N*. *benthamiana* replicating TBSV RNA showed a close to 3-fold enhanced TBSV accumulation (Fig 4F), suggesting that Pgk1 level affects tombusvirus replication. Altogether, these data have confirmed the pro-viral function of Pgk1 in tombusvirus replication in plants.

### Knock-down of Pgk1 level decreases the local accumulation of ATP within the tombusvirus replication compartment

**Fig 5:**
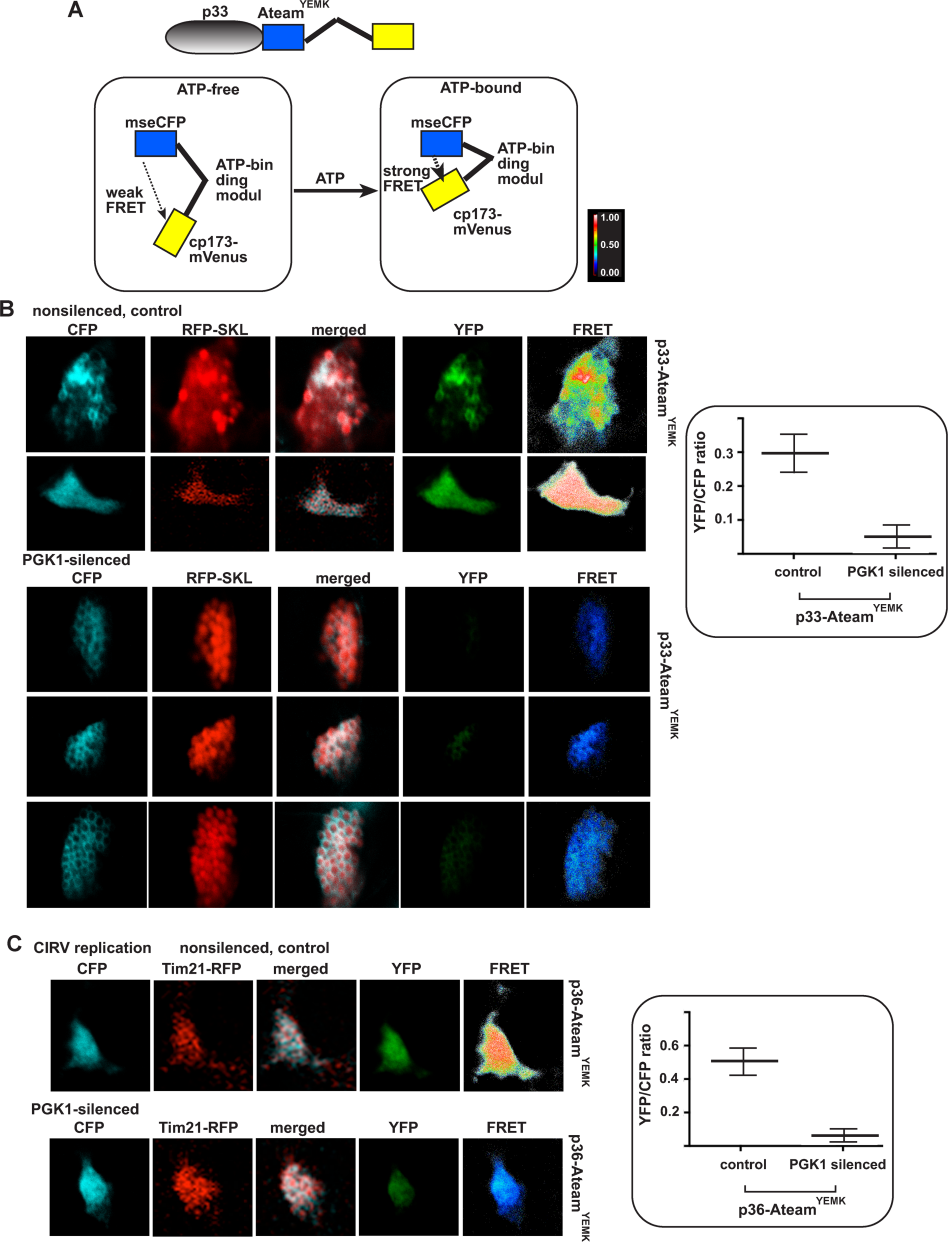
The cytosolic Pgk1 affects ATP accumulation within the tombusvirus replication compartment in *N*. *benthamiana*. (A) A scheme of the FRET-based detection of cellular ATP within the replication compartment. The enhanced ATP biosensor, ATeam^YEMK^ was fused to TBSV p33 replication protein. (B) Knock-down of *PGK1* mRNA level by VIGS in *N*. *benthamiana* was done as in Fig 4. Twelve days later, co-expression of p33-ATeam^YEMK^ and RFP-SKL (peroxisomal luminar marker) was done in upper *N*. *benthamiana* leaves by agroinfiltration. The CFP signal indicates the distribution of p33-ATeam^YEMK^, which co-localizes with RFP-SKL to the aggregated peroxisomes. YFP signal was generated by mVenus in p33-ATeam^YEMK^ via FRET. The FRET signal ratio is shown in the right panels. The more intense FRET signals are white and red (between 0.5 to 1.0 ratio), whereas the low FRET signals (0.1 and below) are light blue and dark blue. We also show the average quantitative FRET values (obtained with ImageJ) for 10–20 samples on the graph. (C) Comparable experiments with *PGK1* knock-down *N*. *benthamiana* using the mitochondrial CIRV p36 replication protein tagged with ATeam^YEMK^ and Tim21-RFP mitochondrial marker protein. See further details in panel B.

Based on the above results, we assumed that the co-opted Pgk1 likely generates ATP within the viral replication compartment. Therefore, we estimated the local ATP level within the replication compartment by using the p33 replication protein tagged with ATeam, a cellular ATP-sensor module. ATeam module can measure ATP levels via FRET due to the conformational change in the enhanced ATP-binding domain of the bacterial ATP synthase upon binding to ATP without ATP hydrolysis (Fig 5A) [31]. In the ATP-bound form, the ATP-sensor module brings the CFP and YFP fluorescent tags into proximity, increasing FRET, which can be detected by confocal laser microscopy. In the ATP-free stage, the extended conformation of the ATP-sensor module places CFP and YFP at distal position, leading to low FRET signal. The ATeam-tagged p33 localizes to the aggregated peroxisomes that represent the sites of replication (Fig 5B).

We found by intracellular expression of p33-ATeam that the local ATP level within the TBSV replication compartment was decreased by ~6-fold in Pgk1 knock-down in *N*. *benthamiana* leaves in comparison with control leaves (Fig 5B). Interestingly, the mitochondria-replicating CIRV also accumulated a ~10-fold lower level ATP within the replication compartment in Pgk1 knock-down leaves than in control leaves (Fig 5C). Therefore, we conclude that Pgk1 is recruited to the sites of tombusvirus replication to generate high concentration of ATP within the replication compartment.

### The co-opted Pgk1 supports viral replicase complex assembly within the tombusvirus replication compartment

**Fig 6:**
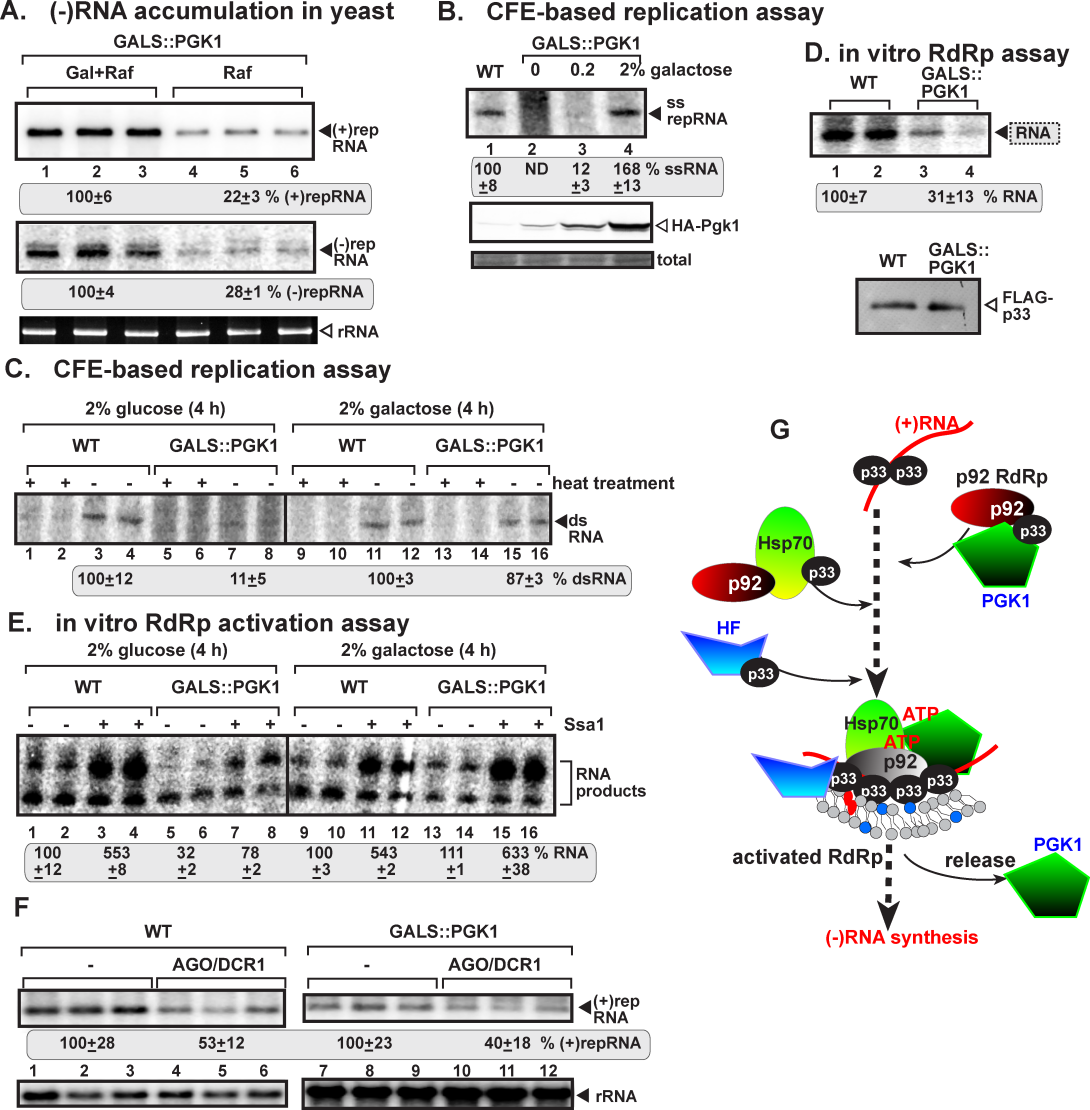
The co-opted function of cytosolic Pgk1 is to provide ATP for viral replicase complex assembly. (A) Northern blot analysis shows decreased TBSV (+) and (-)repRNA accumulation in a yeast strain (GALS::PGK1) when Pgk1 was depleted (raffinose) in comparison when Pgk1 expression is induced (Gal+Raf). See further details in Fig 3, panel A. (B) Inefficient activities of the tombusvirus replicases assembled *in vitro* in CFEs prepared from a yeast strain (GALS::PGK1) with reduced level of Pgk1 expression. Purified recombinant p33 and p92^pol^ replication proteins of TBSV and *in vitro* transcribed TBSV DI-72 (+)repRNA were added to the CFEs prepared from the shown yeast strains. Top panel: denaturing PAGE analysis of *in vitro* tombusvirus replicase activity in the CFEs. The ^32^P-labeled TBSV repRNA products of the reconstituted replicases are shown. Note that the full cycle of replicase activity of the in vitro reconstituted TBSV replicase depends on Pgk1 levels in the CFEs. The CFEs contained the same amounts of total yeast proteins. (C) Reduced production of ^32^P-labeled dsRNA intermediate, consisting of complementary (+) and (-)repRNA strands, by the in vitro reconstituted TBSV replicase when the CFEs were obtained from GALS::PGK1 yeast when Pgk1 was depleted (yeast was cultured in glucose media for 4 h) in comparison when Pgk1 expression is induced (yeast was cultured in galactose media for 4 h). Heat treatment (marked by “+”) was used to show the dsRNA nature of the shown RdRp products. Each experiment was performed three times. (D) Reduced activity of the purified tombusvirus replicase from yeast with depleted Pgk1 level. The membrane-bound replicase complex was collected by centrifugation, followed by solubilization and FLAG-affinity purification from yeasts. Representative denaturing gel of ^32^P-labeled RNA products synthesized by the purified tombusvirus replicase *in vitro*. The *in vitro* assays were programmed with RI/III (-)repRNA, and they also contained ATP/CTP/GTP and ^32^P-UTP. Note that the original viral template RNA in the replicase from yeast is removed during replicase solubilization/purification. (E) In efficient *in vitro* activation of the RdRp function of the N-terminally truncated p92^pol^ replication protein. The soluble fraction of CFEs were obtained from GALS::PGK1 yeast when Pgk1 was depleted (yeast was cultured in glucose media for 4 h) in comparison when Pgk1 expression is induced (yeast was cultured in galactose media for 4 h). Denaturing PAGE analysis of the ^32^P-labeled RNA products obtained in an *in vitro* assay with recombinant p92-Δ167N RdRp. The samples contained or lacked affinity-purified yeast Ssa1p Hsp70 protein (7 pmol). The faster migrating RNA represents a prematurely terminated product, whereas the slower migrating RNA is terminated at the 5’ end of the template. Each experiment was performed three times. (F) Co-expression of *S*. *castellii* AGO1 and DCR1 in GALS::PGK1 yeast (BY4741 background) when Pgk1 was depleted, reduces TBSV repRNA accumulation to a similar extent as in wt yeast (BY4741). Top panel: Replication of the TBSV repRNA was measured by Northern blotting 32 h after initiation of TBSV replication. The accumulation level of repRNA was normalized based on the ribosomal (r)RNA. Each sample is obtained from different yeast colonies. Yeast strain not expressing RNAi components is taken as 100% in each experiment. Average value and standard deviation is calculated from all the biological repeats. Each experiment was repeated twice. Ribosomal RNA is shown as a loading control. (G) A model on the role of the co-opted glycolytic Pgk1 in tombusvirus replication. Direct interaction of the cytosolic Pgk1 with the viral p33 and p92 replication proteins leads to recruitment of Pgk1 into the membranous replication compartment, where Pgk1 generates ATP to fuel the function of the co-opted cellular Hsp70 molecular chaperone. Hsp70 (Ssa1p and Ssa2p in yeast) has been shown to facilitate the assembly of the viral replicase complex, insertion of the replication proteins into peroxisomal membranes and activation of the RdRp function of p92^pol^. It seems that at least a portion of co-opted Pgk1 is released from the active viral replicase complex.

To decipher the function of the co-opted Pgk1 during TBSV replication, we tested viral RNA levels in yeast replicating TBSV repRNA. Down-regulation of Pgk1 expression reduced both (+)- and (-)RNA levels by ~4-fold (Fig 6A). These data suggest that Pgk1 likely plays an early role, prior to viral RNA synthesis, possibly during replicase assembly steps.

To test this scenario, we performed various *in vitro* assays. First, to further examine if Pgk1 was required for viral RNA replication, we obtained cell-free extracts (CFE) from wt yeast and GALS::PGK1 yeast with suppressed Pgk1 expression to reconstitute the TBSV replicase *in vitro* based on purified recombinant TBSV proteins. The yeast CFE-based assay, which supports a single full cycle of viral RNA replication [32], showed ~7-to-10-fold reduced repRNA production when low level of Pgk1 was produced in GALS::PGK1 yeast (Fig 6B, lanes 3 versus 1 and 4, also S4 Fig). Interestingly, both (+)RNA product (Fig 6B) and dsRNA replication intermediate obtained with CFE from GALS::PGK1 yeast with depleted Pgk1 in comparison with the wt yeast CFE were decreased by~7-fold (Fig 6C, lanes 7–8 versus 3–4). Therefore, the *in vitro* replication results support the important role of Pgk1 in viral RNA replication.

We also obtained purified replicase preparations from GALS::PGK1 yeast with suppressed Pgk1 expression versus comparable preparations from wt yeast, which were programmed with viral RNA transcripts [33]. In spite of having comparable amounts of replication proteins, the purified replicase preparations from Pgk1 depleted yeast showed ~3-fold reduced activity in comparison with the similar preparations obtained from wt yeast (Fig 6D).

Since the activity of the purified replicase preparations mostly depends on the efficiency of replicase assembly and replication protein activation, we compared the efficiency of p92^pol^ activation by the supernatant fraction of CFEs prepared from Pgk1 depleted or wt yeasts. These assay revealed ~3-fold less RdRp activity of the purified recombinant p92^pol^ mutant by the preparation from Pgk1 depleted than from wt yeasts (compare lanes 1–2 and 5–6 in Fig 6E). The major active component of the supernatant fraction of CFEs is Hsp70 molecular chaperone, which is essential for the activation of p92^pol^ RdRp in vitro [29]. Accordingly, addition of purified Ssa1 (yeast Hsp70) to the WT fraction has increased the efficiency of p92^pol^ RdRp mutant activation by ~5.5-fold, whereas the comparable assay with Ssa1 involving the preparation from Pgk1 depleted yeast showed only ~2-fold enhancement (compare lanes 3–4 and 7–8 in Fig 6E). Therefore, the *in vitro* results from multiple assays support the model that Pgk1 has a major role in tombusvirus RNA replication and Pgk1 likely fuels the energy requirements of co-opted Hsp70 molecular chaperones to facilitate the efficient replicase assembly and replication protein activation within the viral replication compartment.

TBSV co-opts Vps4p AAA+ ATPase, which is an ATP-dependent host protein, into the viral replicase complex to facilitate replicase assembly [7]. Vps4p is an ESCRT protein involved in membrane deformation, which is necessary for formation of TBSV-induced individual spherules (vesicle-like structures) supporting viral RNA replication [7]. The spherule formation protects the viral dsRNA replication intermediate from the innate RNAi machinery during infection [34]. To test if Pgk1-produced ATP might support Vps4p ATPase function during replicase assembly, we utilized a recently developed intracellular probe based on a reconstituted RNAi system in yeast (*Saccharomyces cerevisiae*), which lacks the RNAi machinery. The reconstituted RNAi machinery from *S*. *castellii*, which consists of the two-component *DCR1* and *AGO1* genes [35], is a simple, and easily tractable system [34]. We found that the induction of RNAi activity had only slightly more inhibitory effect on TBSV RNA accumulation in Pgk1-depleted yeast than in wt yeast (Fig 6F). Based on these results, the structure of the assembled tombusvirus replicase in Pgk1 depleted yeast might not be different from those assembled in wt yeast. Therefore, likely the co-opted ESCRT machinery and the Vps4p ATPase activity might not be affected when Pgk1 is depleted. Alternatively, the residual Pgk1 still present in GALS::PGK1 yeast could provide enough ATP for Vps4p ATPase activity during the replicase assembly process.

## Discussion

TBSV replication, similar to other (+)RNA viruses, is a very intensive and robust process that likely depends on consumption of a large amount of ATP. By recruiting the glycolytic Pgk1 into the virus replication compartment, Pgk1 could efficiently supply ATP to facilitate various steps in the replication process, including the assembly of the viral replicase complex. Accordingly, we show that TBSV actively recruits the cytosolic Pgk1 to the sites of viral replication through direct interaction with the viral replication proteins in both yeast and plant cells. It seems that a portion of recruited Pgk1 is getting released from the sites of replication when the formation of new viral replicases is halted via inhibition of translation. This observation suggests that the ATP produced by the co-opted Pgk1 is likely used up locally to fuel early steps in virus replication, such as viral replicase assembly.

The proposed role of the co-opted Pgk1 during the early steps in virus replication is further supported by additional observations. For example, down regulation of Pgk1 level in yeast affected both (-) and (+)-strand RNA levels and also decreased the in vitro activity of the purified replicase preparations obtained from yeast. Moreover, the in vitro assembly of functional TBSV replicase in CFEs from yeast with depleted Pgk1 level was inefficient, resulting in reduced production of both dsRNA intermediate and new (+)RNA progeny. All these results point at deficiency in viral replicase assembly when Pgk1 is depleted. We have shown previously that the assembly of the tombusvirus replicase requires the co-opted cellular Hsp70 that uses ATP for its molecular chaperone function [32,36,37]. Based on the above observations, we propose that the ATP generated by the recruited Pgk1 serves the energy need of co-opted Hsp70 to drive efficient replicase complex assembly within the elaborate replication compartment (Fig 6G).

In addition, we obtained evidence that the functional activation of the TBSV p92 RdRp, which also requires Hsp70 molecular chaperone, depends on the ATP generated by the recruited Pgk1. The initially inactive p92 RdRp becomes functional during replicase assembly in the presence of p33 replication protein, the viral (+)RNA, ER membrane and the ATP-dependent Hsp70 [27,29]. In a simplified RdRp activation assay, we found that CFEs obtained from yeast with depleted Pgk1 were inefficient in promoting p92 RdRp activity. The addition of purified recombinant Hsp70 to the above assay could stimulate p92 RdRp activity to lesser extent in case of depleted Pgk1 than in the presence of wt yeast CFEs. Therefore, we propose that a major function of the Pgk1-generated ATP is to provide fuel to the co-opted Hsp70 during p92 RdRp activation and the assembly of viral replicase complexes (Fig 6G). The advantage of co-opting Pgk1 to the replicase complex could be that the energy hungry virus replicase assembly process does not need to intensively compete with cellular processes for access to plentiful ATP. Also, local production of ATP within the replication compartment could greatly facilitate the efficiency of Hsp70-driven replicase assembly by providing high ATP concentration within the replication compartment. There is previous evidence that Pgk1 could provide ATP to enhance the chaperone activity of Hsp90 that leads to multistress resistance [21].

In addition to Hsp70 molecular chaperone, TBSV also co-opts additional ATP-dependent host proteins into the viral replicase complex, including Vps4p AAA+ ATPase, involved in replicase assembly [7], and two DEAD-box helicases [38,39]. Using a reconstituted RNAi-based molecular probe, we found only a slightly less RNA protection in Pgk1-depleted yeast than in wt yeast (Fig 6F), which is in contrast with the poor TBSV RNA protection provided by VRCs assembled in the absence of ESCRT components [34]. Therefore, we propose that Vps4p AAA ATPase still functions efficiently enough in VRC assembly in yeast with depleted Pgk1 and ATP levels. On the other hand, the DEAD-box helicases, however, selectively affect (+)-strand synthesis, while Pgk1 affects both (-) and (+)-strand synthesis in CFE-based assay and in yeast to a similar extent. TBSV also affects the actin network, which requires ATP for functions, but it is currently on open question if the co-opted Pgk1 supplies ATP for the subverted actin network. Therefore, based on the collected data, we propose that the ATP-generated by Pgk1 within the replication compartment is primarily used by tombusviruses to provide ATP for the co-opted Hsp70 chaperone to support efficient assembly of the numerous viral replicase complexes.

Similar to our current findings, cancer cells also use glycolysis to efficiently generate ATP, even in the presence of oxygen [22]. Up-regulation of glycolytic machinery promotes rapidly proliferating cancer cells survival. Up-regulation of glycolytic pathway also takes place in effector T cells and it is required for immune cells activation [20,40]. Apparently, the relatively inefficient glycolytic pathway can provide enough ATP when up-regulated in these cells.

Several viruses are also known to reprogram the glycolytic pathway during infections based on metabolomic profiling [41,42,43], which unraveled enhanced glucose uptake into the infected cells. The hexokinase activity is increased in hepatitis C virus (HCV) or Dengue virus-infected cells [41,44]. ATP was shown to accumulate at the sites of HCV replication, likely to satisfy the energy demand of virus replication [45]. Whereas TBSV exploits glycolytic enzymes, such as PGK1 (this work) and Glyceraldehyde 3-phosphate dehydrogenase (GPDH, called Tdh2p and Tdh3p in yeast) for pro-viral functions [13,15], replication of a plant potexvirus is inhibited by GAPDH due to its RNA-binding function [46]. Altogether, metabolic reprogramming of the glycolytic pathway might be a widespread phenomenon during various viral infections.

In addition to its role in ATP production during glycolysis, Pgk1 seems to have additional roles in virus replication. For example, Sendai virus, a negative-strand RNA virus, co-opts Pgk1 to promote viral mRNA synthesis, albeit its enzymatic activity is not required for the stimulation of RNA synthesis [47]. A partial/recessive resistance in *Arabidopsis* against potyviruses is due to a single amino acid mutation in the conserved N-terminal portion of the chloroplast phosphoglycerate kinase (cPGK2), which is a nuclear gene encoding cPgk2 targeted to the chloroplast [48,49]. In addition, cPgk1 was found to bind to the 3’UTR of the viral RNA and it is involved in the localization of potexvirus RNA to the chloroplasts, which is important for virus accumulation [50,51].

## Materials and methods

### Northern blot analysis of tombusvirus RNA accumulation

TBSV RNA replication in yeasts and plants was analyzed after total RNA extraction with Northern blot analyses as described previously [52]. Briefly, BY4741 and GalS::PGK yeast strains were co-transformed with pHisGBK-CUP1- Hisp33/ADH-DI-72 and pGAD-CUP1-His92 [24]. The transformed yeast strains were grown at 23°C in SC-HL^−^ (synthetic complete medium without histidine and leucine) media supplemented with 2% raffinose with or without 2% galactose and BCS for 24 h at 23°C. Then, yeast cultures were re-suspended in SC-HL^−^ medium supplemented with 2% raffinose and with or without 2% galactose and 50 μM CuSO_4_. Yeasts were grown at 23°C for 16 h before being collected for total RNA extraction.

### Protein analysis by Western blotting

Yeast cells were grown as described above for Northern analysis. Total proteins were isolated by the NaOH method as described previously [53]. The total protein samples were analyzed by SDS-PAGE and Western blotting with anti-His and anti-PGK antibodies, followed by alkaline phosphatase-conjugated anti-mouse secondary antibody (Sigma) as described previously [54].

### Confocal microscopy in plants

To examine the subcellular localization of Pgk1 in plants, *N*. *benthamiana* leaves were co-infiltrated with Agrobacterium carrying plasmids pGD-p92-YFP and pGD-BFP-PGK (OD_600_ of 0.5, each) together with pGD-35S::p19, pGD-DI-72 and pGD-p33. After 48 h, the agroinfiltrated leaves were subjected to confocal microscopy (Olympus America FV1000) using 405 nm laser for BFP and 488 nm laser for YFP. Images were taken successively and merged using Olympus FLUOVIEW 1.5 software.

To identify interactions between NbPgk1 and TBSV p33 replication proteins, the plasmids pGD-p33-cYFP, pGD-nYFP-PGK and pGD-nYFP-MBP were transformed to Agrobacterium strain C58C1. These Agrobacterium transformants were used to co-agroinfiltrate the leaves of four weeks-old *N*. *benthamiana* plants. Transformed leaves were subjected to confocal laser microscopy after 48 h using Olympus FV1000 microscope as described previously [13].

### Visualization and measurement of ATP levels in plants

To detect the ATP levels within the tombusvirus replication compartments in plant cells, in the case of TBSV, PGK-silenced plants or control plants were agroinfiltrated with plasmids pGD-p33-ATeamYEMK, pGD-DI-72, pGD-35S::RFP-SKL, pGD-35S::p19 and pGD-p92. In the case of CIRV, the leaves were agroinfiltrated with pGD-p36-ATeamYEMK, pGD-35S::AtTim21-RFP, pGD-35S::p19, pGD-DI-72 and pGD-p95. The images were taken at 2.5 or 3.5 days post-agroinfiltration and analyzed with the method described previously [31]. Confocal FRET images were obtained with an Olympus FV1000 microscope (Olympus America). Cells were excited by a 405 nm laser diode, and CFP and Venus were detected at 480–500 nm and 515–615 nm wavelength ranges, respectively. Each YFP/CFP ratio was calculated by dividing pixel-by-pixel a Venus image with a CFP image using Olympus FLUOVIEW software or ImageJ software.

Additional standard experimental procedures are presented in the supporting information S1 Text.

## Supporting information

S1 Fig Co-purification of Pgk1 with the p33 viral replication protein. Top panel: Western blot analysis of co-purified His_6_-tagged Pgk1 with FLAG-affinity purified FLAG-p33 from the membrane fraction of yeast. Pgk1 was detected with anti-His antibody. The negative control was FLAG-tagged GFP purified from the soluble (S) and membrane-fractions (M) of yeast extracts using a FLAG-affinity column. Second panel: Western blot of purified FLAG-p33 and FLAG-GFP detected with anti-FLAG antibody. Third panel: Western blot of His_6_-tagged Pgk1 and His_6_-p33 (lane 1) proteins in the total yeast extracts using anti-His antibody. Fourth panel: Western blot analysis of FLAG-p33 and FLAG-GFP in the total yeast extracts with anti-FLAG antibody. Bottom panel: Coomassie Blue-stained SDS-PAGE with total protein extracts is shown as a loading control. Each experiment was repeated two times.
(EPS)

S2 Fig Reduced TBSV repRNA accumulation in yeast with depleted Pgk1 level at a late time point. Northern blot analysis shows decreased TBSV repRNA accumulation in a yeast strain (GALS::PGK1) when Pgk1 was depleted. To launch TBSV repRNA replication, we expressed His_6_-p33 and His_6_-p92^pol^ from the copper-inducible *CUP1* promoter, and DI-72(+) repRNA from the *ADH1* promoter in the parental (BY4741) and in GALS::PGK1 yeast strains. Note that GALS::PGK1 yeast strain expresses HA-tagged Pgk1 from the galactose-inducible *GALS* promoter from chromosomal location (i.e., HA-Pgk1 replaces the wt Pgk1 in this haploid yeast strain). The yeast cells were cultured for 24 hours at 29°C in either 2% galactose +2% raffinose [(Raf+Gal), inducing condition for HA-Pgk1] or 2% raffinose (lack of induction of HA-Pgk1 expression) SC minimal media supplemented with 50 μM CuSO_4_. The accumulation level of DI-72(+) repRNA was normalized based on 18S rRNA levels (second panel, Northern blot).
(EPS)

S3 Fig Knock-down of Pgk1 level by VIGS reduces the symptoms caused by CNV. *N*. *benthamiana* plants were VIGS treated for 9 days, followed by inoculation with CNV. The pictures were taken 10 days after post infection. The control plants were VIGS treated with TRV-cGFP (the C-terminal half of the GFP sequence).
(EPS)

S4 Fig Reduced activity of the TBSV replicase assembled *in vitro* in CFEs prepared from a yeast strain (GALS::PGK1) with reduced level of Pgk1 expression. Purified recombinant MBP-p33 and MBP-p92^pol^ replication proteins of TBSV and *in vitro* transcribed TBSV DI-72 (+)repRNA were added to the CFEs prepared from the shown yeast strains. Top panel: denaturing PAGE analysis of *in vitro* tombusvirus replicase activity in the CFEs. The ^32^P-labeled TBSV repRNA products of the reconstituted replicases were detected by denaturing PAGE. Middle panel: The amount of Pgk1 was estimated by Western blotting using anti-Pgk1 antibody in yeast lysates. The sample from GALS::PGK1 yeast strain is shown on the right. Bottom panel: The CFEs contained the same amounts of total yeast proteins as demonstrated by Coommassie blue-staining of SDS-PAGE.
(EPS)

S1 Text Additional materials and methods used in this study. (DOCX)
