# Supplementary material for: Co-opting ATP-generating glycolytic enzyme PGK1 phosphoglycerate kinase facilitates the assembly of viral replicase complexes
Source: PLoS Pathog. 2017 Oct 23;13(10):e1006689. doi: 10.1371/journal.ppat.1006689 (PMC5695612; doi:10.1371/journal.ppat.1006689)
Supplement: S1 Text — (DOCX) [file ppat.1006689.s005.docx]

**S1 Text.** Additional materials and methods used in this study.

**Yeast strains and expression plasmids.** The yeast (*Saccharomyces cerevisiae*) strain BY4741 (*MAT*a *his3*Δ*1 leu2*Δ*0 met15*Δ*0 ura3*Δ*0*), was obtained from Open Biosystems. *PGK1* expression was placed under regulatable *GALS* promoter (short version of *GAL1* promoter) by homologous recombination in GalS::PGK yeast strain. To create the GalS::PGK yeast strain, the *GALS* promoter along with the nourseothricin resistance gene was amplified from vector pFA6-pYM-N32 (Euroscarf) [1] with *PGK1* start codon. The PCR-amplified product (#6327: (ACAGATCATCAAGGAAGTAATTATCTACTTTTTACAACAAATATAAAACAATGCGTACGCTGCAGGTCGAC) and #6328 (CGCTTGTCCTTCAAGTCCAAATCTTGGACAGACAACTTTGAAGATAAAGACATCGATGAATTCTCTGTCG) was purified by phenol/chloroform and transfected to BY4741 yeast and the transformants with *GALS* promoter inserted in place of *PGK1* native promoter were selected on the nourseothricin plate and confirmed by Northern and Western blotting. The positive GalS::PGK strains were maintained on YPG media. The plasmid UpYC_2_-BFP-PGK was generated by PCR-amplifying the yeast *PGK1* ORF with the following set of primers #6343 (GGACTAGTATGTCTTTATCTTCAAAGTTG) and #6344 (CCGCTCGAGTTATTTCTTTTCGGATAAGAA). BFP ORF was PCR-amplified with the primer pair #5673 and #5674. Both PCR products were digested with SpeIF restriction enzyme and ligated. The BFP-PGK ligation product was PCR-amplified with primers #5673 and #6344. The obtained PCR product was cloned in to UpYC_2 ­_vector digested with BamHI and XhoI.

To obtain the plasmid UpRS316 TEF:His-PGK, the yeast *PGK1* ORF was PCR- amplified with primers #6365 (CGGGATCCATGCATCATCACCATCACCATTCTTTATCTTCAAAGTTG) and #6344 (CCGCTCGAGTTATTTCTTTTCGGATAAGAA), and the obtained PCR product was cloned into UpRS316 vector at BamHI and XhoI sites.

To generate the plant expression plasmids pGD-PGK-His, the plant *NbPGK1* gene was RT-PCR-amplified from the total RNA of *N. benthamiana* with primers #6437 (CGGGATCCATGGCGGTGAAGAAGAGCGTG) and #6439 (CGCGTCGACTCAATGGTGATGGTGATGATGGGCATCATCTAGGGCAAGCA). The obtained PCR-product was digested with BamHI and SalI and cloned into pGD vector at BamHI/SalI sites. To generate plasmids pGD-CFP-PGK and pGD-nYFP-PGK, the *NbPGK1* gene was PCR-amplified with primers #6437 (CGGGATCCATGGCGGTGAAGAAGAGCGTG) and #6438 (CGCGTCGACTCAGGCATCATCTAGGGCAAGCACT). The obtained PCR product was cloned into pGD-CFP plasmid at BglII*I*SalI sites or into BamHI/SalI-digested pGD-nYFP-MBP plasmid. To generate plasmids pGD-p33-ATeam^YEMK^ and pGD-p36-ATeam^YEMK^, the p33 (TBSV) or p36 (CIRV) sequences were PCR-amplified using primers #1974 / #6793 or #6184 / #6795, followed by the digestion of PCR products with BamHI/XhoI and then, cloned into pGD-35S –L vector at BamHI/XhoI sites, resulting plasmid pGD-p33 (no stop codon) or pGD-p36 (no stop codon). The ATeam^YEMK^ fragment was PCR-amplified using primers #1293 and #6806, followed by the digestion of PCR product with XhoI/SalI and then, cloned into XhoI/SalI-digested pGD-p33 or pGD-p36 plasmid, resulting plasmids pGD-p33-ATeam^YEMK^ and pGD-p36-ATeam^YEMK^.

To generate plasmid HpESC-Gal1::Flag-rsGFP, first, the plasmid pGD-35S::GFP was used as template for PCR with primers #5160 (GGACTAGTGAGTCCGGACTTGTATAGTTCA) and #7104 (CGGAATTCATGGGTAAAGGAGAAGAACTTTTCACTGG) to obtain rsGFP fragment. Then the obtained PCR product was cloned into HpESC plasmid at EcoRI/SpeI sites. To generate plasmid pGD-35S::p92-cYFP, the plasmid UpYES-T92 was used as template for PCR with primer #6075 (CGCGGATCCTAAACAATGGAGACCATCAAGAGAATG) and #5838 (GCCCTGCAGAGCTACGGCGGAGTCGAG) to obtain the TBSV p92 fragment. The obtained PCR product was cloned into plasmid pGD-C-cYFP at BamHI/PstI sites.

### Co-purification of Pgk1 protein with the p33/p92 replication proteins from yeasts. For co-purification of Pgk1 protein with the membrane-bound viral replicase, containing p33 and p92 replication proteins [2], yeast strains BY4741 was co-transformed with plasmids HpGBK-CUP1-FLAGp33/GAL1-DI-72, LpGAD-CUP1-FLAGp92 or HpESC-Gal1::Flag-rsGFP and HpGBK-CUP1-Hisp33/Gal1-DI-72, whereas LpGAD-CUP1-Hisp92 was used as a control [2]. This BY4741 strain was also transformed with UpRS316-TEF-PGK plasmid expressing His_6_-tagged Pgk1 protein from the TEF1 promoter. The transformed yeast cells were pre-grown in SC-ULH^−^ media supplemented with 2% glucose and 100 μM BCS at 29°C for 24 h. Then, the yeast cultures were supplemented with 50 μM CuSO_4_ to induce expression of FLAG-p33 and FLAG-p92 or His_6_-p33 and His_6_-p92 from the CUP1 promoter, and yeast cultures were grown for an additional 4 or 6 h at either 29 °C or 32°C. The cultures were centrifuged, washed once with phosphate-buffered saline (PBS), and then incubated in PBS buffer containing 1% formaldehyde for 1 h on ice to cross-link proteins [2]. Then, glycine (to 0.1M) was added to quench the formaldehyde and the yeasts were recovered by centrifugation. Yeast cells were broken with glass beads and p33 and p92 proteins were purified using anti-FLAG M2 agarose [3]. Affinity-purified FLAG-p33 was analyzed by Western blot using anti-FLAG antibody, and co-purified 6xHis-tagged host proteins were analyzed with anti-His antibody.

### To analyze the time dynamics of Pgk1p association with the viral replicase, transformed BY4741 were pre-grown in in SC-ULH^−^ media supplemented with 2% glucose for 20 h at 29°C and then, transferred to SC-ULH^−^ media supplemented with 2% galactose for 24 h at 29°C. The cultures were supplemented with 50 µM CuSO_4_ for 2.5 h to induce expression of the viral proteins. Then, cycloheximide was added (100 µg/ml) to the media to stop protein translation in yeasts and samples were taken at 0, 60 and 150 minute time points. Yeast cultures were treated with formaldehyde and processed for FLAG-affinity purification of the viral replicase as above.

### Confocal Laser Microscopic Analysis in Yeast. To observe the subcellular localization of Pgk1p in the presence or absence of viral components in yeast, BY4741 strain was transformed with plasmids HpESC-GAL1::GFP-p33 (or empty vector as control), UpRS316-TET::BFP-PGK1 and LpRS315-TET::Pex13-RFP. The transformed yeasts were grown in ULH^-^ media supplemented with 2% galactose at 23°C for overnight. The confocal images were obtained sequentially with an Olympus FV1000 microscope (Olympus America) and merged using Olympus FLUOVIEW 1.5 software.

### Confocal laser microscopy in plants. To examine the subcellular localization of phosphoglycerate kinase (Pgk1) in plants, N. benthamiana leaves were co-infiltrated with Agrobacterium carrying the following plasmids pGD-p92-YFP (0.2 OD_600_), pGD-p33(0.2 OD_600_), pGD-DI-72(0.2 OD_600_), pGD-BFP-PGK (0.2 OD_600_), pGD-SKL (0.2 OD_600_) and pGD-p19 (0.2 OD_600_). pGD empty vector was used as control. After 48 h, the agroinfiltrated leaves were subjected to confocal laser microscopy (Olympus America FV1000) using 405 nm laser for BFP and 488 nm laser for YFP. Images were taken sequentially and merged using Olympus FLUOVIEW 1.5 software.

### To identify interactions between NbPgk1 and TBSV p33 and p92 replication proteins, the plasmids p33-cYFP or p92-cYFP, nYFP-PGK and nYFP-MBP were transformed to Agrobacterium strain C58C1. The Agrobacterium transformants were used to infiltrate four weeks old *N. benthamiana* plants. Agroinfiltrated leaves were subjected to confocal laser microscopy after 48 h using Olympus FV1000 microscope as described previously [4].

### Intracellular probing of VRCs with reconstituted RNAi in yeast. BY4741, and GalS::PGK yeast strains were co-transformed with pGBK-CUP1- Hisp33/Adh-DI-72, pGAD-CUP1-His92 and UpESC-Gal1::AGO1/GAL10::DCR1 (or pESC empty vector as control) [5]. The transformed yeast strains were grown at 23°C in SC-ULH^−^ (synthetic complete medium without histidine and leucine) medium supplemented with 2% glucose and 50 µM CuSO_4_ for 24 h. Then, yeast cultures were re-suspended in SC-ULH^−^ medium supplemented with 2% galactose and grown at 23°C for 8 h before being collected for total RNA extraction.

### Yeast cell free extract (CFE)-based in vitro replication assay. CFEs from BY4741 and GalS::PGK1 were prepared as described earlier [6]. Briefly, BY4741 and GalS::PGK1 were pre-grown in YPD and YPG, respectively, at 29°C overnight, then the cultures were transferred to fresh YPD and/or YPG media and grew at 29°C for additional 4 h. The CFEs were prepared as described and adjusted to contain comparable amounts of total protein [6]. The in vitro replication assays were performed in 20 µl total volume containing 1 µl of adjusted CFEs, 0.5 µg DI-72 (+)RNA transcripts, 0.5 µg purified MBP-p33, 0.5 µg purified MBP-p92^pol^ (both recombinant proteins were MBP-affinity purified from E. coli), 30 mM HEPES-KOH, pH 7.4, 150 mM potassium acetate, 5 mM magnesium acetate, 0.13 M sorbitol, 0.4 µl actinomycin D (5 mg/ml), 2 µl of 150 mM creatine phosphate, 0.2 µl of 10 mg/ml creatine kinase, 0.2 µl of RNase inhibitor, 0.2 µl of 1 M dithiothreitol (DTT), 2 µl of 10 mM ATP, CTP, and GTP and 0.1 mM UTP and 0.1 µl of ^32^P-UTP. Reaction mixtures were incubated for 3 h at 25 °C, followed by phenol/chloroform extraction and isopropanol/ammonium acetate (10:1) precipitation. ^32^P-UTP-labeled RNA products were analyzed in 5% acrylamide/8 M urea gels [6].

### In vitro RdRp activation assay. The CFEs prepared from BY4741 and GalS::PGK yeast strains were centrifuged at 4°C for 20 min at 42,000 × g to obtain the soluble (supernatant) fraction. The FLAG-tagged yeast Ssa1p (Hsp70) was over-expressed in yeast and purified using FLAG-affinity purification [7]. The RdRp activation assay was conducted with the soluble fraction of CFEs and MBP-p92Δ167N using 0.5 µg (+)DI-mini RNA template [8]. In additional RdRp activation assay, we added affinity-purified FLAG-Ssa1p (0.5 µg) to observe the RdRp activation function of Ssa1p in different CFEs.

### Over-expression of Pgk1 in *N. benthamiana* leaves. For transient over-expression of NbPgk1 in *N. benthamiana* leaves, we PCR-amplified the PGK1 sequence using the following pair of primers: #6437 (CGGGATCCATGGCGGTGAAGAAGAGCGTG) and #6439 (CGCGTCGACTCAATGGTGATGGTGATGATGGGCA-TCATCTAGGGCAAGCA). The obtained PCR product was digested with BamHI and SalI and ligated into pGD vector. The resulting pGD-PGK-His plasmid was transformed into *Agrobacterium tumefaciens* C58C1. Plants were agroinfiltrated with *A. tumefaciens* carrying pGD-PGK-His or pGD (empty plasmid) and pGD-35S::p19 [9]. One day later, the agroinfiltrated leaves were sap inoculated with TBSV. Two days later, leaf samples were collected from the TBSV-infected leaves and total RNA was extracted, followed by Northern blotting to determine TBSV RNA accumulation level [10].

### VIGS of cytosolic PGK1 in *N. benthamiana* plants. The virus-induced gene silencing (VIGS) in *N. benthamiana* was completed as described previously [10]. To generate the VIGS vector (pTRV2-PGK), a 229-bp cDNA fragment of *N. benthamiana PGK1* was reverse-transcribed (RT) and PCR-amplified from total RNA extract of *N. benthamiana* using primers #6440 (CGGGATCCAGAATCAGAGCCGCTGTACC) and #6441 (GCGAGCTCATTCTCTAGCAGCAGAACTCC). The obtained RT-PCR product was inserted into the corresponding (BamHI and SacI) restriction sites of plasmid pTRV2 [9]. Eight days after the VIGS treatment (agroinfiltration of young leaves with pTRV2-PGK vector together with pTRV1), the level of *N. benthamiana PGK1 (*NbPGK1*)* mRNA was determined by RT-PCR with primers #6442 (CGGGATCCTTACTTGGCGGAGGAATGAT) and #6443 (GCGAGCTCCCAAATGACAGTCTTGGTGGT). We used tubulin mRNA as a control for RT-PCR using primers #2859 (TAATACGACTCACTATAGGAACCAAATCATTCATGTTGCTCTC) and #2860 infected with TBSV, CNV or CIRV. Samples from the infected leaves were collected three days after sap inoculation with TBSV and agroinfiltration with CNV and CIRV, followed by total RNA extraction and Northern blot analysis as described previously [10].

**References:**

1. Janke C, Magiera MM, Rathfelder N, Taxis C, Reber S, et al. (2004) A versatile toolbox for PCR-based tagging of yeast genes: new fluorescent proteins, more markers and promoter substitution cassettes. Yeast 21: 947-962.

2. Barajas D, Martin IF, Pogany J, Risco C, Nagy PD (2014) Noncanonical Role for the Host Vps4 AAA+ ATPase ESCRT Protein in the Formation of Tomato Bushy Stunt Virus Replicase. PLoS Pathog 10: e1004087.

3. Li Z, Barajas D, Panavas T, Herbst DA, Nagy PD (2008) Cdc34p ubiquitin-conjugating enzyme is a component of the tombusvirus replicase complex and ubiquitinates p33 replication protein. J Virol 82: 6911-6926.

4. Wang RY, Nagy PD (2008) Tomato bushy stunt virus co-opts the RNA-binding function of a host metabolic enzyme for viral genomic RNA synthesis. Cell Host Microbe 3: 178-187.

5. Kovalev N, Inaba JI, Li Z, Nagy PD (2017) The role of co-opted ESCRT proteins and lipid factors in protection of tombusviral double-stranded RNA replication intermediate against reconstituted RNAi in yeast. PLoS Pathog 13: e1006520.

6. Pogany J, Stork J, Li Z, Nagy PD (2008) In vitro assembly of the Tomato bushy stunt virus replicase requires the host Heat shock protein 70. Proc Natl Acad Sci U S A 105: 19956-19961.

7. Pogany J, Nagy PD (2012) p33-Independent Activation of a Truncated p92 RNA-Dependent RNA Polymerase of Tomato Bushy Stunt Virus in Yeast Cell-Free Extract. J Virol 86: 12025-12038.

8. Pogany J, Nagy PD (2015) Activation of Tomato Bushy Stunt Virus RNA-Dependent RNA Polymerase by Cellular Heat Shock Protein 70 Is Enhanced by Phospholipids In Vitro. J Virol 89: 5714-5723.

9. Dinesh-Kumar SP, Anandalakshmi R, Marathe R, Schiff M, Liu Y (2003) Virus-induced gene silencing. Methods Mol Biol 236: 287-294.

10. Jaag HM, Nagy PD (2009) Silencing of Nicotiana benthamiana Xrn4p exoribonuclease promotes tombusvirus RNA accumulation and recombination. Virology 386: 344-352.
